# The role of phenotypic plasticity in the establishment of range margins

**DOI:** 10.1098/rstb.2021.0012

**Published:** 2022-03-14

**Authors:** Martin Eriksson, Marina Rafajlović

**Affiliations:** ^1^ Department of Marine Sciences, University of Gothenburg, Gothenburg, Sweden; ^2^ The Linnaeus Centre for Marine Evolutionary Biology, University of Gothenburg, Gothenburg, Sweden; ^3^ Gothenburg Global Biodiversity Centre, University of Gothenburg, Gothenburg, Sweden

**Keywords:** cost of plasticity, critical environmental gradient, range limits, environmental fluctuations, genetic canalization, climate change adaptation

## Abstract

It has been argued that adaptive phenotypic plasticity may facilitate range expansions over spatially and temporally variable environments. However, plasticity may induce fitness costs. This may hinder the evolution of plasticity. Earlier modelling studies examined the role of plasticity during range expansions of populations with fixed genetic variance. However, genetic variance evolves in natural populations. This may critically alter model outcomes. We ask: how does the capacity for plasticity in populations with evolving genetic variance alter range margins that populations without the capacity for plasticity are expected to attain? We answered this question using computer simulations and analytical approximations. We found a critical plasticity cost above which the capacity for plasticity has no impact on the expected range of the population. Below the critical cost, by contrast, plasticity facilitates range expansion, extending the range in comparison to that expected for populations without plasticity. We further found that populations may evolve plasticity to buffer temporal environmental fluctuations, but only when the plasticity cost is below the critical cost. Thus, the cost of plasticity is a key factor involved in range expansions of populations with the potential to express plastic response in the adaptive trait.

This article is part of the theme issue ‘Species' ranges in the face of changing environments (part I)’.

## 1. Introduction

Owing to ongoing climate change and increasing human impact on ecosystems, many populations need to adapt to novel conditions either in their present geographical distributions, or in new areas they face while altering their ranges [1–5]. A critical factor constraining local adaptation and thereby precluding successful range expansions is maladaptive gene flow [6,7]. Theoretically, it has been shown that, when genetic variance is fixed and the population is faced with a sufficiently steep constant environmental gradient, maladaptive gene flow swamps local adaptation. This results in a finite range of the population [8] (see also [9]).

However, genetic variance in natural populations is expected to evolve. Notably, the above theoretical prediction is critically altered when genetic variance is allowed to evolve. Under this assumption, populations expanding their ranges over an environment that changes linearly in space (with a constant carrying capacity) will either adapt to the entire available habitat or face global extinction [10]. In this case, therefore, range margins are trivial: they either coincide with the habitat edges or, when the habitat is unlimited, range margins are absent.

By contrast, non-trivial range margins exist when a population expands its range over a steepening environmental gradient, and this is true even when the available habitat is infinite [10,11]. In this case, local genetic variance increases with increasing local steepness of the environmental gradient until the genetic load becomes so strong that the population is precluded from adapting further. This is seen as a progressively decreasing expected local population size (despite the assumption that the carrying capacity is constant over the habitat) down to the point where drift becomes stronger than selection [11]. Conversely, for range expansions over environments that change linearly in space (with genetic variance allowed to evolve), drift may cause non-trivial range margins to be established when the local carrying capacity decreases away from the core habitat [11].

The results outlined above deliver an insight into potential mechanisms involved in the establishment of range limits. However, they do not account for phenotypic plasticity (hereafter referred to as *plasticity*), that is, the ability of a genotype to produce different phenotypes depending on the environment [12–16]. Plasticity may be an important mechanism for populations to buffer environmental changes, as shown both empirically [17–23] and theoretically [9,13,16,24–26]. This is especially true when plasticity is adaptive (moving phenotypes towards the local optimum) [27,28]. However, plasticity may also be neutral or maladaptive (moving phenotypes away from the local optimum) [29] (see also [30]). Maladaptive plasticity may have a temporary adverse effect on local adaptation but, in the long run, it may promote genetic adaptation by enhancing the strength of selection [31–33].

However, it has been empirically observed that plasticity does not always contribute to the persistence of populations [34]. Indeed, plasticity may have costs or limits [35,36], and these may limit the use of plasticity for adaptation to new or changing environments [37].

Understanding the evolution of plasticity along environmental gradients, and its role on local adaptation, has been the focus of a number of theoretical studies (e.g. [9,24,26,38]). For example, in [24], it was found that, in areas where the difference between the local phenotypic optimum and the globally average optimum was larger, local adaptation was facilitated by the evolution of locally higher plasticity. This is, in part, because migration was implemented according to the island model (*sensu* [39]). In this model, immigration has a strongly deleterious effect on the local mean phenotype when it deviates strongly from the global mean. This causes local maladaptation, which produces directional selection to restore the local mean phenotype to its optimum. Consequently, plasticity is under stronger selection when the difference between the local environment and the reference environment (as defined in [40]) is larger. Notably, the model in [24] was deterministic and it was assumed that genetic variance was fixed. These assumptions may have both qualitative and quantitative consequences for the results obtained.

A similar result was found in a model with an environment that changes linearly in space and a density regulated population (*albeit* without drift) [9]. As a consequence, plasticity increased the range attained by the population in comparison to the case without plasticity [9]. Notably, the results in [9] relied on two assumptions that may critically affect the model outcomes, especially regarding the range that the population is expected to attain. Namely, genetic variance was fixed and the carrying capacity was decreasing away from the centre of the range. As explained above (see also [11]), these assumptions are responsible for the establishment of non-trivial range margins in an environment that changes linearly in space. These assumptions were relaxed in [26], where it was found that transiently increased plasticity evolves in spatial locations that have a long history of environmental change, or at the expansion front for a population undergoing range expansion into a habitat that requires new adaptations (termed ‘niche expansion’ in that study). Notably, in [26], the environment changed linearly in space. This precluded the establishment of non-trivial range margins in that study.

In summary, the role of plasticity on the establishment of non-trivial range margins, when genetic variance is allowed to evolve, remains unclear. Here, we address this issue by modelling a population, with evolving genetic variance, expanding its range over a steepening environmental gradient. This is a situation in which a population without plasticity is expected to attain a non-trivial range margin, even when the carrying capacity is not constrained to be decreasing away from the core habitat [11]. Specifically, we ask: how does a population’s capacity for plasticity impact on the establishment of range margins when genetic variance is allowed to evolve and the local carrying capacity is constant? What is the role of plasticity costs in this context? What is the spatial pattern of allele frequencies at the underlying loci?

To answer these questions, we extend the individual-based model from [11] to encompass the capacity for plasticity. This was done by assuming that the adaptive trait had a non-plastic and a plastic component. We further used a simplified version of our model to derive an analytical expression for the *optimal plasticity*, that is, plasticity that maximizes the population’s mean fitness in quasi-equilibrium. We note that we used here *quasi*, because all finite populations with a finite growth rate will eventually go extinct [41]. With this caution in mind, we use throughout *equilibrium* in place of *quasi-equilibrium*, for simplicity.

Our main finding is that there is a critical cost of plasticity below which the ability to express and evolve plasticity leads to a wider range than for populations lacking this ability. Furthermore, we found a second critical cost below which the range may be infinite. Finally, we found that the equilibrium spatial patterns of allele frequencies at loci contributing to the non-plastic component of the phenotype have the same clinal shape as without plasticity, but the spacing between the clines is increased when plasticity is larger. For the plastic component of the phenotype, we found that the frequencies of alleles associated with positive plasticity increased in a cline-like manner towards the edges of the habitat only when the cost of plasticity was below the critical cost. Otherwise, no clinal pattern emerged.

## 2. Methods

We used computer simulations to investigate the impact of plasticity on the evolution of range margins. The simulations were performed using custom-made Matlab code (available from [42]).

We extended the model previously considered in [43] (see also [11,44]), in which a population expanded its range over a habitat with a steepening environmental gradient, assuming a single trait under selection. In addition, in the present work, we assumed that the phenotype was determined by a combination of a non-plastic and a plastic component. We further allowed the optimal phenotype to fluctuate in time. These model modifications are explained in more detail below.

The habitat consisted of a one-dimensional chain of *M* = 220 demes, each with a local carrying capacity of *K* = 100 diploid individuals (unless otherwise stated; see the electronic supplementary material, appendix A for details regarding parameter choices, and table 1 that lists the notations used throughout). The generations were discrete and non-overlapping. The individuals were monoecious and mating was assumed to occur randomly with selfing allowed at no cost. As in [11,43], we assumed a gradually steepening environmental gradient along the habitat: in each deme, *i* = 1, 2, …, *M*, the average optimal phenotype for the trait under selection, ${\bar{\theta }}^{\left(i\right)}$, was given by a cubic polynomial of the deme number, *i*, such that ${\bar{\theta }}^{\left(i\right)}$ ranged between ±252.9 (electronic supplementary material, figure A1). This polynomial was chosen to be symmetric with a horizontal inflection point at the centre of the habitat, where the optimal phenotype was assumed to be zero (electronic supplementary material, appendix A). Recall that a steepening (but not a constant) environmental gradient allows non-trivial range margins to be established in a population lacking the capacity for a plastic response. To further understand the role of a gradually steepening as opposed to a constant gradient on the evolution of the spatial pattern in plasticity in the population, we also performed simulations along an environment that changes linearly in space (i.e. along a constant gradient; electronic supplementary material, appendix A). We further assumed that the realized optimal value for the phenotype is either temporally constant or that it fluctuates in time. In the latter case, we assumed that in deme *i* in generation *τ*, the optimal phenotype (denoted by $\theta_{\tau }^{\left(i\right)}$ hereafter) is a normally distributed random variable with mean ${\bar{\theta }}^{\left(i\right)}$ and standard deviation *σ*_*θ*_ (see the electronic supplementary material, table A1 for a list of parameter values explored). For simplicity, we assumed that fluctuations in the optimal phenotype were temporally and spatially uncorrelated.

**Table 1.** Explanation of the notations used throughout.

notation	description
*M*	number of demes in the habitat
*K*	carrying capacity per deme
$N_{\tau }^{\left(i\right)}$	local population size in deme *i* in generation *τ*
$\theta_{\tau }^{\left(i\right)}$	optimal phenotype in deme *i* in generation *τ*
${\bar{\theta }}^{\left(i\right)}$	average optimal phenotype in deme *i*
*σ*_*θ*_	standard deviation of fluctuations in the optimal phenotype
$u_{\tau ,k}^{\left(i\right)}$	phenotype of the trait under selection for individual *k* in deme *i* in generation *τ*
$z_{\tau ,k}^{\left(i\right)}$	non-plastic component of the phenotype for individual *k* in deme *i* in generation *τ*
$g_{\tau ,k}^{\left(i\right)}$	plasticity of the phenotype for individual *k* in deme *i* in generation *τ*
$W_{\tau ,k}^{\left(i\right)}$	fitness of individual *k* in deme *i* in generation *τ*
$C_{\gamma }(g_{\tau ,k}^{\left(i\right)},\delta )$	cost-related function for plasticity
*γ*	shape parameter for the cost-related function
*δ*	scale parameter for the cost-related function
$r_{\tau ,k}^{\left(i\right)}$	growth rate of individual *k* in deme *i* in generation *τ*
*r*_*m*_	maximal intrinsic growth rate
*V*_*S*_	width of stabilizing selection
*μ*	mutation rate
*L*	number of loci under selection for the non-plastic as well as for the plastic component of the phenotype (total of 2*L* loci)
±*α*/2	effect size of alleles coding for the non-plastic component of the phenotype
±*β*/2	effect size of alleles coding for plasticity
*s*	selection per locus for loci underlying the non-plastic component of the phenotype, *s* = *α*^2^/(2*V*_*S*_)
*σ*	standard deviation of Gaussian dispersal function

We assumed that the phenotype, $u_{\tau ,k}^{\left(i\right)}$, of the trait under selection for individual *k* in deme *i* in generation *τ* was equal to the sum of a non-plastic and a plastic component:

2.1 $$u_{\tau ,k}^{\left(i\right)}=z_{\tau ,k}^{\left(i\right)}+g_{\tau ,k}^{\left(i\right)}\theta_{\tau }^{\left(i\right)},$$

where $z_{\tau ,k}^{\left(i\right)}$ denotes the non-plastic component and $g_{\tau ,k}^{\left(i\right)}$ denotes the magnitude of the individual’s plastic response relative to the local phenotypic optimum (hereafter referred to as *plasticity*). The full plastic component of the phenotype was assumed to be equal to $g_{\tau ,k}^{\left(i\right)}\theta_{\tau }^{\left(i\right)}$, reflecting a common assumption (e.g. [25,26]) that the same environmental variable determines both the plastic response and the optimal phenotype. For simplicity, we use $\theta_{\tau }^{\left(i\right)}$ to denote both the optimal phenotype and the environmental cue that affects the plastic response. Note that ${\bar{\theta }}^{\left(i\right)}$ was zero in the centre of the habitat, hence plasticity had, on average (i.e. ignoring the temporal fluctuations), no effect on the average phenotype there. This setting corresponds to treating the centre of the habitat (which is the source of expansion in the model) as the *reference environment* for the plastic response [40]. Note that equation (2.1) corresponds to eqn (2) in [26] in the special case when the reference environment, *g*_2_ in the notation from [26], is zero.

In our model, the non-plastic component of the phenotype, $z_{\tau ,k}^{\left(i\right)}$, and plasticity $g_{\tau ,k}^{\left(i\right)}$, were each underlain by *L* freely recombining bi-allelic loci with additive allele effects, that is, in total there were 2*L* loci under selection (but we also performed simulations where the number of loci for the plastic and non-plastic component were different; electronic supplementary material, appendix C). The two possible allele effect sizes for the loci underlying $z_{\tau ,k}^{\left(i\right)}$ were ±*α*/2 with $\alpha =({\bar{\theta }}^{\left(M\right)}/L)$ so that in the absence of plasticity (i.e. when $g_{\tau ,k}^{\left(i\right)}=0$), the *L* loci underlying $z_{\tau ,k}^{\left(i\right)}$ were just enough to constitute the average minimal and maximal optimal phenotypes in the habitat, i.e. the optima at the habitat edges (analogously to [43]). The two possible allele effect sizes for the loci underlying $g_{\tau ,k}^{\left(i\right)}$ were ±*β*/2 with *β* = 2/*L* so that $g_{\tau ,k}^{\left(i\right)}$ was between −2 and 2. In a special case when $g_{\tau ,k}^{\left(i\right)}=1$ and $z_{\tau ,k}^{\left(i\right)}=0$, it follows that $u_{\tau ,k}^{\left(i\right)}=\theta_{\tau }^{\left(i\right)}$. Noting that the optimal phenotype in the source of the expansion is, on average, zero, we refer to plasticity of one (i.e. $g_{\tau ,k}^{\left(i\right)}=1$) as *perfect plasticity*, because it allows perfect adaptation everywhere without any evolution of the non-plastic component with respect to the source of the expansion.

Apart from assuming that plasticity had a polygenic basis, we also allowed it to be potentially costly. Namely, we modelled the fitness $W_{\tau ,k}^{\left(i\right)}$ of individual *k* in deme *i* in generation *τ* as

2.2 $$W_{\tau ,k}^{\left(i\right)}=2\exp\left(r_{\tau ,k}^{\left(i\right)}\right)C_{\gamma }(g_{\tau ,k}^{\left(i\right)},\delta ).$$

In equation (2.2), the factor 2 is included due to diploidy, $r_{\tau ,k}^{\left(i\right)}$ is the growth rate and $C_{\gamma }(g_{\tau ,k}^{\left(i\right)},\delta )$ is a cost-related function accounting for a maintenance cost of plasticity (*sensu* [35]), such that costs are larger when $C_{\gamma }(g_{\tau ,k}^{\left(i\right)},\delta )$ is smaller, and *vice versa*. These components are further explained next.

The growth rate, $r_{\tau ,k}^{\left(i\right)}$, was assumed to be given by

2.3 $$r_{\tau ,k}^{\left(i\right)}=r_{m}(1-\frac{N_{\tau }^{\left(i\right)}}{K})-\frac{{\left(u_{\tau ,k}^{\left(i\right)}-\theta_{\tau }^{\left(i\right)}\right)}^{2}}{2V_{S}}.$$

Here, *V*_*S*_ denotes the width of stabilizing selection and we assumed throughout that *V*_*S*_ = 2. Furthermore, *r*_*m*_ denotes the maximal intrinsic growth rate and it was set to *r*_*m*_ = 1 in our simulations. Finally, $N_{\tau }^{\left(i\right)}$ denotes the population size in deme *i* in generation *τ*, and $u_{\tau ,k}^{\left(i\right)}$ denotes the phenotype, given by equation (2.1). Note that when $g_{\tau ,k}^{\left(i\right)}=0$ and *σ*_*θ*_ = 0, the model reduces to the one considered in [43]. Our model did not contain any residual component of phenotypic variance caused by environmental factors in addition to the variability in $\theta_{\tau }^{\left(i\right)}$.

We assumed that $C_{\gamma }(g_{\tau ,k}^{\left(i\right)},\delta )$ is a decreasing function of the absolute value of plasticity $\left|g_{\tau ,k}^{\left(i\right)}\right|$ (similarly as in [26]), that is:

2.4 $$C_{\gamma }(g_{\tau ,k}^{\left(i\right)},\delta )={\left(1-\delta |g_{\tau ,k}^{\left(i\right)}|\right)}^{\gamma }.$$

In equation (2.4), *δ* and *γ* are non-negative parameters, assumed to be constant over time and the same for all individuals. The parameter *δ* determines the threshold plasticity above which the maximal fitness of an individual is non-positive. When $|g_{\tau ,k}^{\left(i\right)}|=1/\delta$, it follows that $C_{\gamma }(g_{\tau ,k}^{\left(i\right)},\delta )=0$, and hence $W_{\tau ,k}^{\left(i\right)}=0$. To avoid occurrences of negative fitness, we define $W_{\tau ,k}^{\left(i\right)}=0$ when $|g_{\tau ,k}^{\left(i\right)}|\geq 1/\delta$. Conversely, the parameter *γ* is a shape parameter determining whether plasticity costs are more sensitive to high or low plasticity. When *δ* = 0 and/or *γ* = 0, it follows that $C_{\gamma }(g_{\tau ,k}^{\left(i\right)},\delta )=1$, and thus there is no cost of plasticity. The cost of plasticity increases with increasing *δ* and/or *γ* (keeping $g_{\tau ,k}^{\left(i\right)}$ constant). A graphical illustration of the cost-related function $C_{\gamma }(g_{\tau ,k}^{\left(i\right)},\delta )$ for *γ* = 1 and *γ* = 0.5 is shown in the electronic supplementary material, figure A2 in appendix A.

The life cycle of individuals was modelled as follows. First, each individual contributed a random number of gametes sampled from a Poisson distribution with mean $W_{\tau ,k}^{\left(i\right)}$ (equation (2.2)). Plasticity was expressed during the adult life stage in the same environment where the individuals mated. Recombination occurred independently for each gamete, with free recombination between all loci. Second, at each locus mutation occurred reversibly and symmetrically between the two possible alleles with probability *μ* = 10^−6^ per allele, per gamete, per generation. Third, pairs of gametes were chosen uniformly at random to form zygotes (thus, selfing was possible). Finally, the parents were removed and the zygotes dispersed according to a Gaussian function with mean 0 and standard deviation *σ* = 1, as described in [43]. After migration, zygotes were treated as adults.

At the start of each simulation, a fraction of the habitat was occupied, and we initialized genotypes in such a way that the average phenotype of the population followed the local optimum in the occupied demes and all individuals initially had plasticity of zero (electronic supplementary material, appendix A). Consequently, the (narrow-sense) heritability [45] of the phenotype was initially set to 1.

After initializing the starting genotypes, we simulated a burn-in period of 100 000 generations in the source population before we allowed expansion over the empty demes. The burn-in period allowed us to initiate range expansion from an old source population. During the burn-in period the source population stabilized under migration, selection, mutation, drift and possible interactions between the plastic and non-plastic component of the phenotype. This reduced the impact of our choice regarding the starting genotypes (described in the electronic supplementary material, appendix A) on the follow-up dynamics of range expansion.

During the burn-in period, the population was restricted to *M*/5 demes in the centre of the habitat. The boundaries were reflecting, that is, individuals remained at boundary demes instead of dispersing out of the initial range. Note that the number of migrants reaching the boundaries was finite in every generation because all demes have a finite number of individuals prior to migration, and dispersal distance was relatively small (*σ* = 1).

After the burn-in, the population was allowed to expand its range for an additional 100 000 generations (or 200 000 generations in some cases; electronic supplementary material, appendix A). As during the burn-in period, the habitat had reflecting boundaries. We examined different parameter sets, chosen below, close to, or above the critical cost of plasticity derived in the electronic supplementary material, appendix B (electronic supplementary material, table A1, appendix A). For each deme, we recorded the population size, the average non-plastic component, the average plasticity, and the genetic variance every 200 generations. The genotype of each individual was recorded at the end of the simulations and at the end of the burn-in period. We performed 100 independent realizations for each parameter set (unless stated otherwise).

Apart from performing simulations, we analytically estimated plasticity that maximizes the mean population fitness locally (i.e. the *optimal plasticity*; electronic supplementary material, appendix B). Notably, we derived approximate conditions for when a population with the capacity for plasticity is expected to attain a larger range than a population lacking this capacity.

## 3. Results

### (a) Analytical approximation of the optimal plasticity and the critical cost of plasticity

To derive the conditions under which the capacity for plasticity may increase the equilibrium range of a population, we have undertaken the following steps. First, we found that a locally optimal plasticity, $g_{e}^{*}$ (*x*) at position *x* (i.e. plasticity that maximizes the local mean population growth rate in equilibrium), in a temporally static environment with a given local environmental gradient *b*(*x*) = ∂*θ*(*x*)/∂*x*, is given by:

3.1 $$g_{e}^{*}(x)=\left\{ \begin{array}{ll} 0,\; & \mathrm{for}\;\frac{1}{\delta }-\frac{2\gamma \sqrt{V_{S}}}{\sigma b(x)}\leq 0,\;\\ 1,\; & \mathrm{for}\;\frac{1}{\delta }-\frac{2\gamma \sqrt{V_{S}}}{\sigma b(x)}\geq 1,\;\\ \frac{1}{\delta }-\frac{2\gamma \sqrt{V_{S}}}{\sigma b(x)},\; & \mathrm{for}\;0<\frac{1}{\delta }-\frac{2\gamma \sqrt{V_{S}}}{\sigma b(x)}<1.\; \end{array}\right.$$

Thus, in spatial regions where the environmental gradient is sufficiently shallow (for $b(x)\leq 2\gamma \delta \sqrt{V_{S}}/\sigma$), the fitness of the population is maximized when the phenotype is entirely determined by its non-plastic component. Conversely, in regions where the environmental gradient is sufficiently steep (for *δ* < 1 and $b(x)\geq 2\gamma \delta \sqrt{V_{S}}/[\sigma (1-\delta )]$), the fitness of the population is maximized when the phenotype is entirely determined by plasticity. Intermediate values of plasticity are favoured for intermediate steepness of the environmental gradient (either for *δ* > 1 and $2\gamma \delta \sqrt{V_{S}}/\sigma <b(x)$ or for *δ* < 1 and $2\gamma \delta \sqrt{V_{S}}/\sigma <b(x)<2\gamma \delta \sqrt{V_{S}}/[\sigma (1-\delta )]$). In temporally fluctuating environments, the optimal plasticity is typically larger than in temporally static environments (electronic supplementary material, equation (B41)). Here, we explain the implications of the optimal plasticity in the case of static environments, for simplicity, but the same arguments apply to temporally fluctuating environments.

Using equation (3.1), we found a critical environmental gradient (hereafter called the *critical plasticity gradient*), below which the optimal plasticity is zero (i.e. when $b(x)\leq 2\gamma \delta \sqrt{V_{S}}/\sigma$). That is, below the critical plasticity gradient any potential positive plasticity that may evolve during initial phases of range expansion is transient, and will eventually vanish.

Next, we made use of the critical plasticity gradient to deduce the conditions allowing a population expanding its range over a gradually steepening gradient to use plasticity. Recall that, for a population without the capacity for plasticity, local adaptation is expected to fail at a critical environmental gradient [11] (hereafter *critical genetic gradient*, to emphasize that it corresponds to the case where plasticity is absent). We conclude that when the critical genetic gradient is smaller than the critical plasticity gradient, local adaptation for a population with the capacity for plasticity fails under the same conditions as for a population lacking the capacity for plasticity.

**Figure 1.**
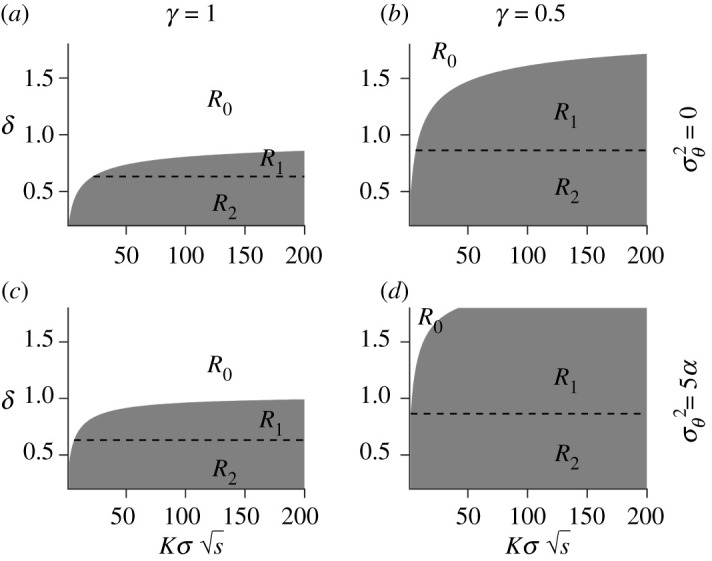
For a given variance of temporal fluctuations in the optimal phenotype, the cost of plasticity divides the parameter space, consisting of the two compound parameters $K\sigma \sqrt{s}$ (the square root of the efficacy of selection per locus underlying the non-plastic component of the phenotype relative to the strength of drift at the carrying capacity) and *γδ*/*r*_*m*_ (the product of the cost-related parameters relative to the maximal intrinsic growth rate), into three regimes, *R*_0_, *R*_1_ and *R*_2_. In regime *R*_0_ (shown in white), range margins form under the same conditions as without plasticity. In regimes *R*_1_ and *R*_2_ (shown in grey), the range is larger than without plasticity. The dashed line corresponds to a maximum mean population growth rate of zero when the mean phenotype is at the optimum and plasticity equals one. Above the dashed line, in regime *R*_1_, the equilibrium range is finite. In regime *R*_2_ (below the dashed line in the grey area) the growth rate of the population is positive for plasticity of 1. Left column: regimes for a linear cost-related function. Right column: regimes for a concave cost-related function (*γ* = 0.5). Upper row: regimes for a temporally static environment. Lower row: regimes for temporally fluctuating environment where $\sigma_{\theta }^{2}=5\alpha$ (with $\alpha =1/\sqrt{10}$).

More generally, we show that there are three different regimes for the range margins (figure 1) with respect to two compound parameters, that is *γδ*/*r*_*m*_ (the product of the two cost-related parameters, *γ* and *δ*, relative to the maximal intrinsic growth rate), and $K\sigma \sqrt{s}$ (the square root of the efficacy of selection per locus underlying the non-plastic component of the phenotype relative to the strength of drift at the carrying capacity; table 1). The three different regimes are: no difference in the range compared to when the population does not have the capacity for plasticity (this regime, hereafter denoted by *R*_0_, is discussed above and corresponds to the white region in figure 1); a larger, but finite, range than when the population does not have the capacity for plasticity (grey region in figure 1, above the dashed line; hereafter denoted by *R*_1_); and potentially infinite range (figure 1, below the dashed line; hereafter denoted by *R*_2_).

Finally, we found a critical cost of plasticity (*δ*_*c*_) below which the critical genetic gradient is larger than the critical plasticity gradient. In other words, the critical cost of plasticity is the smallest cost of plasticity for which the dynamics of range expansion fall within regime *R*_0_. The critical cost of plasticity, generalized to account for temporal fluctuations of environmental conditions (electronic supplementary material, appendix B), is given by

3.2 $$\begin{array}{r} \delta_{c}=\frac{1}{\gamma }(r_{m}\frac{2A+2-AF-\sqrt{4+8A+4AF+A^{2}F}}{2A}+\frac{\sigma_{\theta }^{2}}{\sigma_{\theta }^{2}+V_{S}}). \end{array}$$

Here, $A=0.3\sqrt{2}K\sigma \sqrt{s}$ and $F=-\ln\left[\sqrt{V_{S}/(\sigma_{\theta }^{2}+V_{S})}\right]$ (for notations see table 1). The critical cost (equation (3.2)), separates the white region from the grey in figure 1.

Outside of the parameter region where regime *R*_0_ is realized, i.e. when the cost of plasticity is lower than the critical cost, the equilibrium range of the population is expected to be larger than for a population without the capacity for plasticity. Here, the equilibrium range is either finite, but larger than for a population without the capacity for plasticity (*R*_1_) or it is possibly infinite (*R*_2_; note that regime *R*_2_ accounts for cases where unlimited ranges occur, but this may not happen for all parameters belonging to regime *R*_2_, as we discuss next).

We distinguished regimes *R*_1_ and *R*_2_ using a necessary but not sufficient condition for unlimited range expansion (dashed line in figure 1), namely that the cost of plasticity is both lower than the critical cost *δ*_*c*_, and sufficiently low to allow a positive population growth rate with plasticity of 1 (hereafter *perfect plasticity*; electronic supplementary material, appendix B).

We did not determine the precise conditions allowing unlimited range expansion. However, this is expected at least when there is no cost of plasticity (equations (2.1)–(2.3)). We used simulations to examine several parameter sets belonging to regime *R*_2_, focusing on cases with positive plasticity costs.

### (b) Simulation results

For comparison, we first ran simulations without plasticity (electronic supplementary material, figure C1). In simulations without plasticity and with static environmental conditions ($\sigma_{\theta }^{2}=0$), range margins established at the critical genetic gradient (electronic supplementary material, figure C1 A), as expected. By contrast, temporal fluctuations in the optimal phenotype (in the absence of plasticity) reduced the range by reducing the equilibrium population size by approximately $\ln(\sqrt{V_{S}/(V_{S}+\sigma_{\theta }^{2})})/r_{m}$ in agreement with [46,47] (electronic supplementary material, figure C1 B–D; appendix B). Next, we present simulation results with plasticity.

#### (i) Temporally static environmental conditions

Recall that our simulations were initialized with a burn-in period. When there were no temporal fluctuations in the environmental conditions, the average plasticity at the end of the burn-in period was close to zero (electronic supplementary material, figure C2). As a consequence, the starting genotype for the non-plastic component was essentially the same as without plasticity (electronic supplementary material, figure C3). Although most alleles for plasticity were fixed, some loci were polymorphic (electronic supplementary material, figure C4).

**Figure 2.**
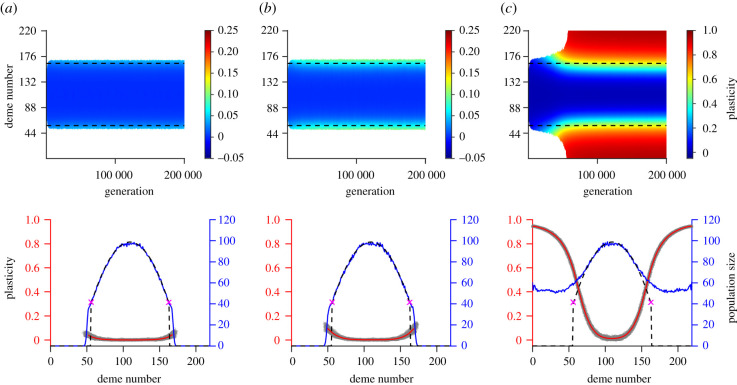
The upper panels show the temporal and spatial evolution of plasticity averaged over 100 realizations during range expansion in a habitat with temporally static environmental conditions. The range expansion dynamics is expected to fall within regime *R*_0_ (column (*a*)), *R*_1_ (column (*b*)) or *R*_2_ (column (*c*)). The columns differ by the parameter *δ*: *δ* = 1.3 (*a*), *δ* = 0.9 (*b*) *δ* = 0.5 (*c*). The red lines in the bottom panels show plasticity averaged over 100 realizations (red axis on the left), the grey areas indicate the spread of plasticity between different realizations. The blue lines show the population size, averaged over 100 realizations (blue axis on the right). The dashed lines in the upper panels denote where adaptation is expected to fail for a population without plasticity. The dashed lines in the lower panels show the expected population size in the absence of plasticity and the purple crosses indicate where adaptation is expected to fail. Remaining parameters: *K* = 100, *r*_*m*_ = 1, *V*_*S*_ = 2, *μ* = 10^−6^, *σ* = 1, *L* = 799, *α* = 0.3162, *β* = 0.0013, *γ* = 0.5 and $\sigma_{\theta }=0$.

After the burn-in period, we found that when the cost of plasticity was higher than the critical cost *δ*_*c*_ (so that the expected range expansion dynamics was within regime *R*_0_), plasticity was very low (<0.05), and the final range agreed with the expected range for populations without the capacity for plasticity (figure 2*a*; electronic supplementary material, figures C5 A,B, C6 A,B). This finding was retained when the cost of plasticity was close to the critical cost (electronic supplementary material, figures C5 C and C6 C).

Conversely, when the cost of plasticity was lower than the critical cost, but sufficiently high to prevent a population with perfect plasticity to have a positive growth rate (i.e. parameters within the expected regime *R*_1_), we observed a higher plasticity in the edges and a slightly larger range than when the cost was above the critical cost (figure 2*b*). For a more concave cost-related function, the difference between the ranges attained in regime *R*_0_ and *R*_1_ was larger (compare electronic supplementary material, figures C7 to figure 2*b*).

By contrast, when the cost of plasticity was both lower than the critical cost and sufficiently low to allow a population with perfect plasticity to have a positive growth rate, the entire habitat was colonized (figure 2*c*; electronic supplementary material, figures C5 D, C6 D).

Recall that our analytical results (equation (3.1)) shows that selection favours fully non-plastic (plastic) phenotypes in shallow (steep) environmental gradients. This is in agreement with our simulations (red lines in the bottom panels in figure 2). Regardless of the cost, during the entire simulated time-span, plasticity remained close to zero in the centre of the habitat, where the environmental gradient is shallow. In the edges of the range, plasticity was higher than in the centre of the range. Furthermore, plasticity in the range edges was higher for parameter combination within regime *R*_1_ than for parameter combinations within regime *R*_0_ (average plasticity was 0.02 in figure 2*a*, in comparison to 0.1 and 0.7 in figure 2*b* and electronic supplementary material, figure C7 A, respectively). For parameters in regime *R*_2_, the entire habitat was populated and plasticity was close to 1 at the habitat edges (0.95 on average in the case shown in figure 2*c*).

The spatial pattern of allele frequencies for the non-plastic component of the phenotype consisted of a series of staggered clines with the same average width as expected for a population without the capacity for plasticity (electronic supplementary material, figure C8). However, when non-zero plasticity evolved, the spacing between the clines was larger than it would have been in the absence of plasticity (e.g. note the absence of clines between deme 10 and deme 50 in the electronic supplementary material, figure C8 A, and compare to the electronic supplementary material, figure C8 C and E). This is expected by the analogy with [10] (*albeit* in a model without plasticity) because plasticity *g* effectively reduces the environmental gradient by a factor of 1 − *g* [24]. Thus, the spacing between the clines is expected to be increased by a factor of 1/(1 − *g*). For the loci underlying plasticity, allele frequencies increased in a cline-like manner towards the habitat edges for parameters within regime *R*_2_ (electronic supplementary material, figure C8 B) and regime *R*_1_ (electronic supplementary material, figure C8 D). By contrast, when no plasticity evolved, no clear spatial pattern in allele frequencies emerged for the loci underlying plasticity (electronic supplementary material, figure C8 F).

#### (ii) Temporally fluctuating environmental conditions

When the model included temporal fluctuations in the optimal phenotype, results similar to those for static environmental conditions were obtained at the end of the burn-in period when the cost of plasticity was above the critical cost (electronic supplementary material, figure C9 A, B and D). However, positive plasticity evolved during the burn-in period when the cost of plasticity was low (electronic supplementary material, figure C9 C, E, F, G, H and I). These results are in agreement with the electronic supplementary material, equation (B41) (and see [48]). The spatial patterns of allele frequencies for the non-plastic component at the end of the burn-in period were more noisy than under temporally static environmental conditions (compare electronic supplementary material, figures C10 to C3). As for temporally static environmental conditions, the spatial pattern of allele frequencies for the plastic component were irregular (electronic supplementary material, figure C11).

**Figure 3.**
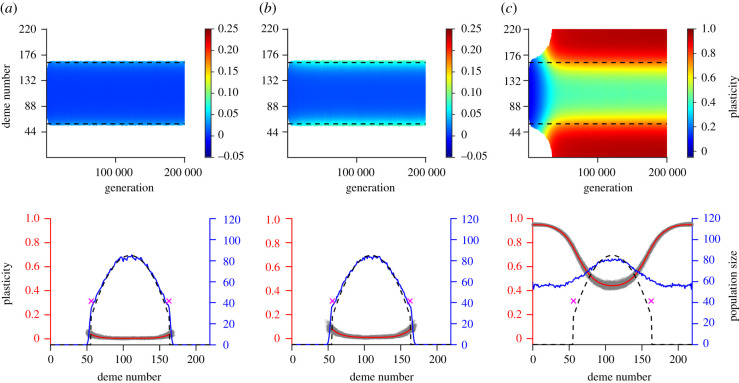
The columns show the results corresponding to those in figure 2 but for temporally fluctuating environmental conditions ($\sigma_{\theta }=\sqrt{2\alpha }$). For the parameter values used (apart from $\sigma_{\theta }$), refer to the caption of figure 2. The dashed lines in the upper panels denote where adaptation is expected to fail (when $\sigma_{\theta }=\sqrt{2\alpha }$) for a population without plasticity. The dashed lines in the lower panels show the expected population size with temporally fluctuating environmental conditions for a population without plasticity (and purple crosses indicate where adaptation is expected to fail for a population without plasticity in a temporally static environment). Remaining parameters: *K* = 100, *r*_*m*_ = 1, *V*_*S*_ = 2, *μ* = 10^−6^, *σ* = 1, *L* = 799, *α* = 0.3162, *β* = 0.0013 and *γ* = 0.5.

After the burn-in period, when the population was allowed to expand its range, no plasticity evolved when the cost of plasticity was larger than the critical cost (figure 3*a*; electronic supplementary material, figures C12 A, C and E; C13 A, C and E), similarly to when the environment was static. In addition, the population size and range extent attained at the end of our simulations were the same as for a population without the capacity for plasticity (compare electronic supplementary material, figures C1 B to C13 A; figures C1 C to C13 C; and figures C1 D to C13 E). Conversely, and similarly to the case with static environmental conditions, when the cost of plasticity was below the critical cost, positive plasticity evolved. For parameters within regime *R*_1_, as expected, the range was larger than in the absence of plasticity, but smaller than the size of the available habitat (figure 3*b*). Conversely, for parameters within regime *R*_2_ very high plasticity evolved (on average, 0.95 at the habitat edges in the case shown in figure 3*c*) and range expansion continued all the way to the edges of the habitat (figure 3*c*; see also the electronic supplementary material, figures C12 B, D, F and C13 B, D, F).

In contrast to the results with temporally static environments, plasticity in the centre of the habitat was close to zero only when the cost of plasticity was high (red lines in the bottom panels of figure 3*a*,*b* and in the electronic supplementary material, figure C13 A, C, E), and it was well above zero in the other cases (red lines in the bottom panel of figure 3*c* and in the electronic supplementary material, figure C13 B, D, F). Thus, a gradient in plasticity at the end of our simulations was shallower with temporally fluctuating than with temporally static conditions (compare figures 2*c*–3*c*). Interestingly, at the end of our simulations with temporally fluctuating environmental conditions, plasticity in the centre of the habitat was higher than at the end of the burn-in period (compare, for example, electronic supplementary material, figure C9 C to figure 3*c*), and higher than the optimal plasticity given by our approximation electronic supplementary material, (B41). This resulted in a lower population size in the centre of the habitat than the population size expected for a population without plasticity.

## 4. Discussion

Plasticity may facilitate local adaptation to variable and marginal environments, as demonstrated empirically (e.g. [49,50]), and theoretically (e.g.[9,24–26,37,40,48,51]). However, in some cases, the impact of plasticity on local adaptation may be weak or non-existent (e.g.[15,27,34,52]). The extent to which plasticity is involved in local adaptation may impact on the evolution of species’ ranges and range margins. However, theoretical understanding of the role of plasticity in the establishment of range margins was limited to situations in which genetic variance is an (arbitrarily) fixed, rather than an evolving, property of a population [9] (but see [26]). Importantly, studies of range expansion in the absence of plasticity [8,10,11] have shown that genetic variance is a key factor involved in the establishment of range margins. Indeed, fixed genetic variance can cause non-trivial range margins to establish (giving rise to finite ranges, smaller than the size of the available habitat), whereas evolving genetic variance, under otherwise the same model conditions, can allow unlimited range expansion [10]. This suggests that allowing genetic variance to evolve, instead of keeping it fixed, may alter the role of plasticity in the establishment of range margins, both qualitatively and quantitatively. This is the focus of our study. We are primarily interested in situations where populations without plasticity would attain non-trivial range margins, such as range expansions over gradually steepening spatial environmental gradients, either without or with temporal fluctuations.

### (a) When does the capacity for plasticity increase the range of a population?

Our main result is that plasticity may be involved in the establishment of range margins in one of the following three qualitatively different ways: (i) no effect of plasticity, (ii) plasticity increases the range by a finite amount, or (iii) plasticity allows for unlimited ranges (i.e. absence of non-trivial range margins). Which of these possibilities is realized depends on the benefits of plasticity relative to its costs. Notably, we found a critical cost of plasticity, *δ*_*c*_, above which plasticity does not evolve and the population (despite the capacity for plasticity) is expected to attain the same range as a population lacking the capacity for a plastic response. Below this cost, the range of the population is wider than the range of a population that lacks the capacity for plasticity. Interestingly, the critical plasticity cost is smaller in temporally fluctuating than in static environments, in agreement with [48]. Furthermore, we found a second (smaller) critical cost (hereafter *threshold cost*) below which the range may be infinite (or constrained by a finite habitat size).

When the cost of plasticity is above the critical cost *δ*_*c*_, in local populations up to and beyond the critical genetic gradient (found in [11]), fitness is maximized when plasticity is zero. As a consequence, above the critical plasticity cost, the equilibrium range of a population with the capacity for plasticity coincides with the range of a population lacking this capacity. This is confirmed by our simulation results. Throughout the range, local plasticity was zero on average, except in local populations in the close vicinity of the range margins where slightly positive plasticity evolved. This is expected because marginal populations are demographic sinks (*sensu* [53]). Here, a strongly positive feedback between local maladaptation and small local population size increases local selection for plasticity [9]. Importantly, however, this effect is weak above the critical plasticity cost, making plasticity ineffective to increase the range beyond the range expected in the absence of plasticity.

By contrast, when the cost of plasticity is below *δ*_*c*_, positive plasticity is optimal below the critical genetic gradient. This allows positive plasticity to evolve and be maintained in local populations. In turn, positive plasticity reduces local maladaptation, as well as local selection gradient (as also suggested in [24]), thus making it possible for a population to expand beyond the range expected in the absence of plasticity (i.e. beyond the critical genetic gradient). Interestingly, when the cost of plasticity is so low that the population may simultaneously express perfect plasticity and have a positive growth rate (i.e. below the threshold cost we found), there may be no limit to range expansion (but note that the threshold cost corresponds to a necessary, but not sufficient condition for infinite range expansion to occur). While we were not able to formally prove that infinite range expansion occurs when plasticity costs are sufficiently small, but positive (note that zero costs trivially result in infinite range expansion, as also pointed out in [9], and see references therein), our simulations with non-zero plasticity costs below the threshold cost confirmed that the population occupied the entire habitat (which is necessarily finite in simulations), and that large plasticity evolved (close to 1 at the habitat edges).

Conversely, when the cost of plasticity is below *δ*_*c*_, but still so large that a population with perfect plasticity cannot have a positive growth rate (i.e. above the threshold cost), the capacity for plasticity leads to a range that is finite but larger than when plasticity is absent. Notably, the width of the parameter region where this regime is realized (i.e. between the critical and the threshold cost) is governed by the concavity of the cost function. The more strongly concave the cost function is, the wider is the regime where plasticity leads to finite but larger ranges than when plasticity is absent. For linear or convex cost functions, this regime is very narrow and almost non-existent for biologically plausible parameters. Consequently, in populations with linear or convex plasticity cost functions, plasticity in equilibrium tends to be either zero throughout the range of the population, or the population may expand its range without limits. We discuss the consequences of this finding in the next subsection.

Our approximation for the optimal plasticity (equation (3.1)) relies on a number of simplifying assumptions in comparison to the simulation model (e.g. we assumed that plasticity is locally constant and that there is no between-individual variation in plasticity; further details in the electronic supplementary material, appendix B). Despite this, the optimal plasticity agrees well with plasticity evolved by the end of our simulations in cases with large effect sizes of the alleles underlying plasticity, but the agreement is poorer with smaller effect sizes of the alleles underlying plasticity (results not shown). This is because when selection is weak relative to dispersal, the allele frequencies at the loci underlying plasticity may change slower in space than required for the mean plasticity of the population to track the optimal plasticity (as suggested in [54] *albeit* in a model without plasticity). Specifically, the optimal plasticity is typically slightly higher than the realized plasticity, except when the optimal plasticity is zero, in which case the realized plasticity is also zero.

Recall that we assumed a gradually steepening spatial environmental gradient. Under this assumption, we found a spatial gradient in plasticity when the cost of plasticity was below *δ*_*c*_. This is similar to the pattern found in e.g. [9,24]. However, in those studies, genetic variance was fixed. Consequently, in [9,24] the mean population phenotype deviated more from the local optimum further away from the core habitat, resulting in an increased selection for plasticity away from the core habitat. In our model, by contrast, genetic variance is allowed to evolve, meaning that the mean population phenotype in populated areas matches the (average) optimal phenotype. Here, maladaptation is owing to genetic variance that increases as the environmental gradient steepens (and this may be further amplified by the local variance in plasticity). This increase in genetic variance is further reflected in a progressively decreasing realized population size (although all demes had the same carrying capacity). Thus, in our model, genetic variance increases as the distance from the core population increases, and this is the main cause of stronger selection for plasticity in our model. This seems consistent with a recent study [26] of range expansion over a habitat with environmental conditions that change linearly in space (i.e. with a constant rather than a steepening gradient) and where genetic variance was allowed to evolve: the authors of [26] argued that a spatial gradient in plasticity levels out in the long run. To examine this further, we performed range expansion simulations along an environment that changes linearly in space (electronic supplementary material, figure C14). We noted a small increase in plasticity towards the habitat edges. This is probably, in part, owing to the mechanism suggested in [9,24], i.e. that local selection for plasticity is stronger when the difference in the local environmental conditions from the reference environmental conditions is larger (despite a constant environmental gradient). However, the (shallow) plasticity gradient emerging in our simulations with a constant environmental gradient (electronic supplementary material, figure C14) may also, in part, be caused by the finite number of loci used in the simulations (this effect is likely to decrease upon increasing the number of loci, but we did not test this further). Importantly, and as expected from our analytical analysis, we found that the gradient in plasticity was much shallower when the environmental gradient was constant (electronic supplementary material, figure C14) than when it was steepening (figure 2*c*). This supports our conclusion that the key factor governing local plasticity is the local environmental gradient (equation (3.1)). In other words, the plasticity gradient emerging in our model is mainly driven by the underlying spatially steepening environmental gradient. However, we note that the plasticity gradient occurs only below the critical plasticity cost.

Finally, in our simulations plasticity evolved slower during range expansion than the non-plastic component of the phenotype. This is both owing to the steepening environmental gradient, which was shallow in the centre of the habitat, and owing to the relatively small allele effect sizes at loci underlying plasticity. By contrast, plasticity evolved much faster in [26], where the environment changed linearly in space and fewer loci were underlying plasticity (so that the allele effect sizes at loci underlying plasticity were larger). Indeed, in our simulations with larger allele effect sizes at loci underlying plasticity (electronic supplementary material, figure C15), or with a constant, rather than steepening, environmental gradient (electronic supplementary material, figure C14), plasticity evolved faster.

### (b) Plasticity costs: empirical data and a lesson from theory

We have analytically re-derived the theoretically well-known result that in the absence of costs, perfect plasticity will eventually evolve [9,55], and the population would be able to expand its range infinitely. The existence of finite ranges even in the absence of any evident geographical barriers [6,7], thus, suggests that some limits or costs of plasticity may be involved [35]. However, empirical evidence for plasticity costs have so far been elusive [36,56,57], except for a few special cases, such as learning-ability [57]. Our results imply that finding empirical evidence for plasticity costs may be specifically difficult when cost functions are much more sensitive to high values of plasticity than to low values (i.e. when cost functions are concave). This is because plasticity would be only weakly costly when plasticity is low or moderate. However, plasticity would still be limited, because high plasticity would exert high costs potentially causing a local population to shrink in size (see discussion above). Thus, concave cost functions of plasticity may potentially limit plasticity while rendering costs difficult to detect. Based on this, we speculate that plasticity costs are more likely to be concave than convex in natural populations, but this is yet to be formally demonstrated.

We note that our results are based on the assumption that the cost of plasticity is constant over space and time. If plasticity costs can evolve, they may decrease over time. However, whether the costs of plasticity will eventually vanish remains an open question for future work.

### (c) Limitations of the model

The impact of plasticity on local adaptation may be limited by unreliable environmental cues [58–60]. Because plasticity may be expressed during different life stages of an organism [61], a mismatch between the environment experienced during development of the plastic response and the environment experienced during selection can occur [35]. In this case, high plasticity during the juvenile life stage may produce a population that is overfitted to the temporal environment, and hence ill adapted to future fluctuations in the environmental conditions. It has been shown both theoretically [40,48,51,58–60,62] and empirically [63] that this may impede the evolution of plasticity. Note, however, that the expression of plasticity may occur once during a short critical life-stage or reversibly throughout the life of an individual [64,65]. The cost of unpredictable cues may be less pronounced for reversible plasticity (compared to when plasticity is irreversible), but this depends on the cost for producing the plastic responses, if such costs are present [62]. In our model, we assumed that the environment of development was perceived without noise and that it was the same as the environment of selection. We leave for future studies to investigate how unreliable cues contribute to the formation of range margins.

Recall that we assumed that all loci recombine freely. Thus, we did not explore the effect of reduced recombination between the loci underlying the non-plastic and/or the plastic component of the phenotype. Dispersal in a spatially heterogeneous environment generates linkage disequilibria between loci, which may lead to maladaptive associations between alleles. This may, in turn, promote the evolution of increased recombination [66]. However, the opposite may be true in marginal habitats [43,67,68]. Indeed, locally beneficial combinations of alleles may be partially protected from maladaptive gene flow if the recombination rate between adaptive loci is low. This may allow populations to persist along environmental gradients steeper than the critical genetic gradient [43]. Reaching gradients above the critical genetic gradient may allow the population to evolve plasticity even when its cost is above the critical cost. Hence, reduced recombination may potentially allow the evolution of higher plasticity in the range margins than when recombination between the adaptive loci is free. However, reduced recombination between loci underlying plasticity and loci underlying non-plastic local genetic adaptation may cause trade-offs that limit the utility of plasticity [69]. Additionally, reduced recombination may possibly lead to more frequent evolution of maladaptive plasticity owing to poor purging of alleles coding for maladaptive plasticity. We leave for further studies to investigate the role of recombination in the evolution of plasticity, and how recombination and plasticity interact to form range margins.

### (d) Management and conservation applications

It is well known that ongoing global climate change is expected to cause directional changes in environmental conditions [70]. However, climate change may also be reflected in stronger temporal fluctuations of environmental conditions in many areas [71]. Management and conservation efforts aimed at mitigating the impact of global climate change should therefore include knowledge and predictions on how temporal fluctuations affect the evolution of natural populations. Specifically, we found that unpredictable conditions may lead to decreased ranges of populations that lack the capacity for plasticity in the trait under selection, or for which the capacity for plasticity in the trait under selection is too costly. By contrast, the ranges of populations that have the capacity for plasticity with a sufficiently low cost may not suffer any adverse effect from environmental fluctuations (unless the correlation between the environment of development and the environment of selection is weak, as discussed above, or the fluctuations are so strong that the population goes extinct before it can evolve sufficient plasticity). Indeed, temporal fluctuations may promote the evolution of plasticity to such an extent that future range expansion may be facilitated in comparison to when the environmental conditions are static. This is only true, however, when the cost of plasticity is sufficiently low, as our results show. More generally, our results show how the key parameters, including the carrying capacity, the maximal intrinsic growth rate, and plasticity costs, jointly impact on the conditions a population may adapt to and tolerate. Notably, we show that enhancing the growth rate or the carrying capacity of a population may potentially facilitate the evolution of plasticity and thereby increase the range of conditions a population may endure. We, therefore, suggest that the parameters identified in our analytical treatment, notably the carrying capacity, the maximal intrinsic growth rate, and plasticity costs should be taken into account, for example, when designing assisted evolution programmes aimed at increasing the tolerance of populations to future climate change [72–75].

Furthermore, invasive species are a major threat to biodiversity worldwide [76,77]. Invasive species often exhibit higher plasticity than non-invasive species do [78–80] and it has been suggested that plasticity may be a key factor governing invasion success [78,80,81]. Here, we emphasize that a key factor may, instead, be the cost of plasticity for the trait under selection relative to the critical cost of plasticity. Thus, management of ecosystems aimed at preventing the spread of invasive species should take plasticity and, specifically, the key parameters involved in determining the critical cost of plasticity (for example, the local carrying capacity, as discussed in the previous paragraph) into account [82–84]. This will be particularly important for mitigating potentially elevated risks of biological invasions associated with climate change [82,85].

## Data accessibility

Computer code is available from the Dryad Digital Repository: https://doi.org/10.5061/dryad.zw3r22880 [42].
